# Simultaneous degeneration of myenteric plexuses and pelvic parasympathetic colonic nerve in slow transit constipation: A case report

**DOI:** 10.1097/MD.0000000000006390

**Published:** 2017-03-24

**Authors:** Zhiqiang Cheng, Kun Zhao, Dongsong Bi

**Affiliations:** a Department of General Surgery, Qilu Hospital, Shandong University, Jinan; b Department of Medicine, No. 89 Hospital of PLA, Weifang, China.

**Keywords:** electron microscopy, parasympathetic nerves, pelvic nerves, slow transit constipation

## Abstract

**Rationale:**

Slow transit constipation (STC) is a common disease of which the etiology is still not clear. Multiple hypotheses have been proposed to explain STC, including autonomic neuropathy, disorders of the enteric nervous system and so forth. Morphological abnormalities of the enteric nerves of the colon in patients with STC have been extensively reported, while there have been no morphological reports focusing on extrinsic extramural fibers from the pelvic plexus to the distal colon (i.e., pelvic parasympathetic colonic nerve) in patients with STC.

**Patient concerns:**

Whether morphological changes of pelvic parasympathetic colonic nerve coexist with abnormalities of the enteric nerves of the colon in the patient with STC.

**Diagnosis:**

Slow transit constipation (STC).

**Interventions:**

The patient with STC underwent a partial colectomy (sigmoid colon and partial descending colon). The fibers of the myenteric plexuses within the removed colon and the myelinated fibers of the pelvic parasympathetic colonic nerve were observed under optical and electron-microscope.

**Outcomes:**

The fibers of the myenteric plexuses showed vacuolated degeneration between the muscularis propria layer under optical microscope. Myelinated fibers of the pelvic parasympathetic colonic nerve showed obvious vacuolated degeneration under electron-microscopic examination.

**Lessons:**

Such a simultaneous neuropathy in both myenteric plexuses and extrinsic extramural nerves has not been documented previously. Our finding supports the notion that neuropathy remains the most plausible explanation for STC, in which nerve dysfunction might occur by way of a degenerative process.

## 1 Introduction

Intestinal motility is regulated in part by the autonomic nervous system. The nervous control occurs on 2 different levels: through the extrinsic system, consisting of sympathetic and parasympathetic nerve fiber, and through the intrinsic system, composed of the intramural intestinal nervous plexuses of the gut wall. There have been numerous studies reporting abnormalities in the myenteric plexus of the colon in patients with slow transit constipation (STC).^[1–3]^ However, no studies have been performed regarding the pelvic parasympathetic colonic nerve. Herein, in the present study, for the first time, we describe a simultaneous pathological change of both myenteric plexuses and pelvic parasympathetic colonic nerve in 1 STC patient.

## 2 Case presentation

A 64-year-old Chinese female, suffering from constipation for more than 10 years, had a defecatory frequency of once per 7 to 10 days. The patient had a history of taking laxatives for 5 years, and was diagnosed of STC^[4]^ based on the medical history as well as colonic transit-time test using radiopaque markers.^[5]^ Barium defecography and rectal balloon expulsion tests had been performed to exclude pelvic outlet disorders and megacolon.^[6,7]^ The patient underwent a partial colectomy (sigmoid colon and partial descending colon) and was discharged routinely. The study was approved by the Ethics Committee of Qilu Hospital of Shandong University.

## 3 Pathological examination

**Figure 1:**
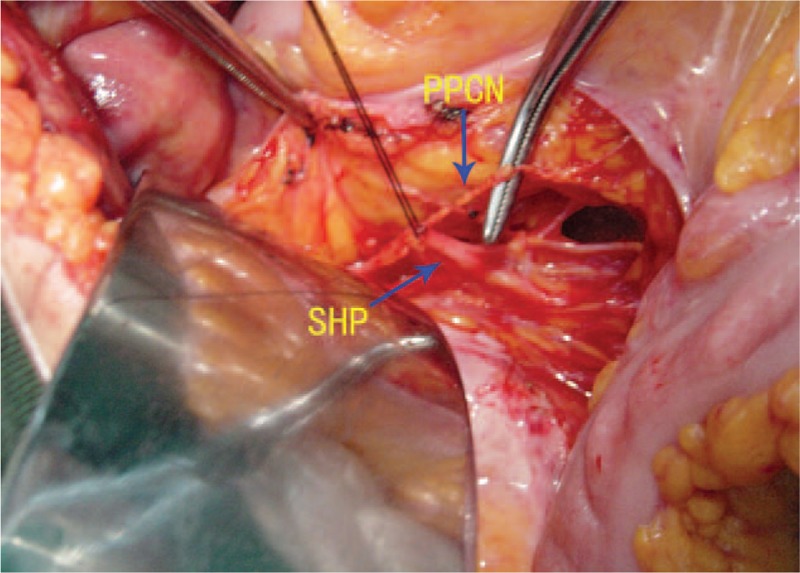
Nerve samples were taken from the pelvic parasympathetic colonic nerve. PPCN = pelvic parasympathetic colonic nerve, SHP = superior hypogastric plexus.

**Figure 2:**
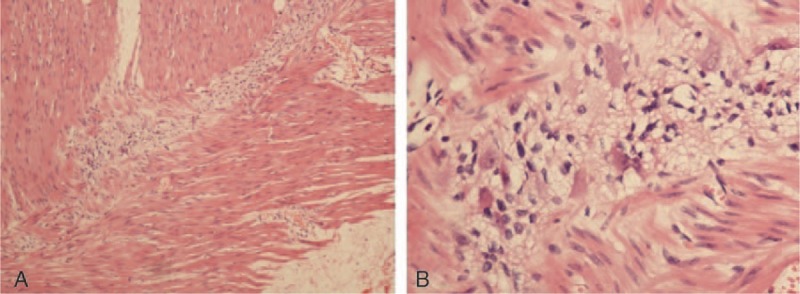
Vacuolated degenerated nerve fibers are diffusely scattered throughout the muscularis propria layer. A, HE stain, ×100. B, HE stain, ×400.

**Figure 3:**
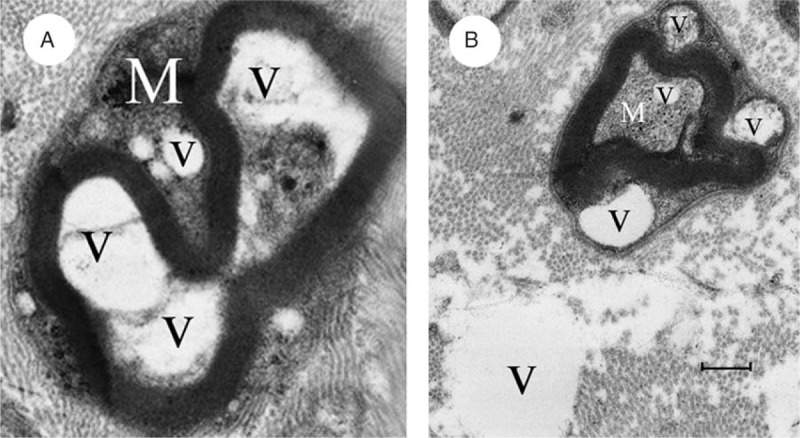
A, Vacuolated areas (V) frequently observed within the myelinated axon (M) and the cytoplasm of the Schwann cells. B, Vacuolated areas (V) external to the Schwann cells (×10,000; bar = 500 nm).

The removed sigmoid colon was taken for routine hematoxylin-eosin staining. The pelvic parasympathetic colonic nerves resected along with the sigmoid colon (Fig. 1) were prepared for electron-microscope examination as described in our previous study.^[8]^ The fibers of the myenteric plexus within the removed colon showed vacuolated degeneration between the muscularis propria layer (Fig. 2). The myelinated fibers of the pelvic parasympathetic colonic nerve exhibited distinct pathological changes under electron-microscopic examination, with vacuolated areas frequently seen within the myelinated axon as well as the cytoplasm of Schwann cells (Fig. 3). Vacuolated areas were also observed external to the Schwann cells.

## 4 Discussion

STC, a functional colonic disorder, represents 15% to 30% of constipated patients.^[9,10]^ It has been recognized that STC is a heterogeneous disease with unknown etiology. Hypotheses proposed to explain STC mostly focused on the disorders of relevant nerves, the enteric nervous system particularly. For example, Zhang et al^[3]^ found the vacuolated degeneration of axons in the myenteric plexuses within the removed colon, which is also consistent with the present report. Apart from the enteric nerves, the pelvic parasympathetic colonic nerves also play an important role in controlling colonic motor function, and have been suspected as another etiological factor of STC. However, there is little direct anatomical evidence.

A number of patients develop STC after pelvic surgery^[11]^ or childbirth,^[12]^ both of which might lead to injury of pelvic parasympathetic nerves. This could be an indirect evidence of pelvic parasympathetic colonic nerves being a contributory factor of STC. For STC patients with no history of potential nerve injury, known as “idiopathic” and also the case in the present report, whether pelvic parasympathetic nerves play a role remains to be elucidated. To our knowledge, we first show the simultaneous degeneration of both pelvic parasympathetic colonic nerves and the enteric nerves, with vacuolated degeneration being a primary pathological change, and provide evidence, though not high-hierarchy evidence, for the notion that dysfunction of pelvic parasympathetic colonic nerves might also be involved in the pathogenesis of STC.

## 5 Conclusion

Aside from the enteric nervous system, vacuolated degeneration is also observed in the pelvic parasympathetic colonic nerve in STC, indicating that neuropathy remains the most plausible explanation for STC, in which nerve dysfunction might occur by way of a degenerative process.
